# Factors influencing the participation of groups identified as underserved in cervical cancer screening in Europe: a scoping review of the literature

**DOI:** 10.3389/fpubh.2023.1144674

**Published:** 2023-05-25

**Authors:** Rachel Greenley, Sadie Bell, Samuel Rigby, Rosa Legood, Victoria Kirkby, Martin McKee, the CBIG-SCREEN Consortium

**Affiliations:** ^1^Centre for Global Mental Health, London School of Hygiene and Tropical Medicine, London, United Kingdom; ^2^Department of Global Health and Development, London School of Hygiene & Tropical Medicine, London, United Kingdom; ^3^Department of Health Services, Research and Policy, London School of Hygiene and Tropical Medicine, London, United Kingdom; ^4^Centre for Global Chronic Conditions, London School of Hygiene & Tropical Medicine, London, United Kingdom

**Keywords:** cervical cancer screening, vulnerable and underserved populations, barriers, facilitators, Europe

## Abstract

**Background:**

Cervical cancer is a preventable and inequitably distributed disease. Screening plays a vital role in prevention, but many women face barriers to participation. The aims of this scoping review, undertaken to inform the co-design of interventions to equitably increase screening uptake, were to: (1) identify barriers and facilitators to cervical cancer screening for underserved populations, and (2) identify and describe the effectiveness of interventions aimed at improving participation in cervical cancer screening among underserved groups in Europe.

**Methods:**

Qualitative, quantitative, and mixed methods studies focusing on barriers and facilitators to cervical screening participation and interventions to improve uptake undertaken in Europe and published after 2000 were included. Four electronic databases were searched to identify relevant papers. Titles and abstracts were screened, full text reviewed, and key findings extracted. Data were extracted and analyzed according to different health system strata: system-wide (macro), service specific (meso) and individual/community specific (micro). Within these categories, themes were identified, and the population groups impacted were recorded. All findings are presented in accordance with (PRISMA) guidelines.

**Results:**

33 studies on barriers and facilitators and eight intervention studies met the inclusion criteria. Collectively, the findings of these studies presented a wide array of screening uptake barriers, facilitators, and interventions, predominantly related to screening service and individual/community factors. However, although diverse, certain core themes around information provision, prompts for participation and the need for inclusive spaces were apparent. Implementation of screening programs should focus on: (1) reducing identifiable barriers, (2) increasing public awareness, and (3) providing patient reminders and measures to promote engagement by healthcare providers.

**Conclusion:**

There are many barriers to uptake of cervical cancer screening and this review, nested within a larger study, will inform work to devise a solution alongside groups identified in three European countries.

## Introduction

Cervical cancer remains the fourth most common cancer in women, even though it is preventable (1). In the mid-1980s, when cancer rates across the European Union (EU) were increasing, Europe Against Cancer launched an ambitious program aiming to achieve a 15% reduction in cancer mortality by the year 2000 (2). It included elements of screening and education, with a focus on actions individuals can take to reduce their risk of developing cancer. Initially limited to guidance on good practice, it developed into an action plan to strengthen prevention of cancer and improve early detection and treatment. Yet, despite the priority that the EU has given to preventing cervical cancer, it still kills many women in Europe (1).

Further progress depends on success in fully implementing programs of vaccination against human papillomavirus (HPV) and cervical cancer screening (CCS), both highly effective and core elements of WHO and EU strategies (3–6). Fortunately, all EU member states now have programs in place to provide vaccines to young women, beginning in 2006, and, as of December 2021, young men (except in the three Baltic Republics, Poland, Bulgaria, Romania, and Greece). The programs were first implemented in 2007 (France, Germany, and 3 regions of Spain) and were extended until 2018 (Estonia). Three products are licensed for use in the EU: a bivalent vaccine (Cervarix), a quadri-valent recombinant vaccine, (Gardasil) and a 9-valent vaccine (Gardasil 9) (7). Yet vaccine coverage is often suboptimal, a problem exacerbated by the narrow time window for vaccination to be effective before initiation of sexual activity and the relatively recent implementation of vaccination programs in some countries, leaving many older women unprotected (8). Thus, effective and accessible screening programs will continue to be needed for some time. However, many screening programs also struggle to cover those in need, leaving large numbers of women, and those with cervixes, vulnerable (9).

Concerning the distribution of cervical cancer across European society, data limitations inhibit insights into the scale and nature of inequalities (10). However, a body of evidence does point to large differences in cervical cancer outcomes and care across population groups. One extensive compilation of data, covering 18 countries, found a three-fold difference, increasing to eight-fold in Estonia, in cervical cancer mortality according to education status, adversely affecting those with low educational attainment. The authors of this study noted how the variation in mortality among countries was driven entirely by differences in outcomes for the least educated women (11).

Examination of the distribution of factors known to prevent mortality, namely vaccination and screening, at country level within Europe, provides further insights into cervical cancer inequalities. In Denmark, a country with especially high quality data, vaccine uptake was found to be lower among girls from minority ethnic groups (odds ratio [OR] = 0.49; 95% confidence interval [CI], 0.42–0.57), mothers with only basic education (OR = 0.75; 95% CI, 0.69–0.82), those with low disposable income (OR = 0.67; 95% CI, 0.61–0.73) and mothers who are unemployed (OR = 0.75; 95% CI, 0.69–0.82) or unmarried (OR = 0.70; 95% CI, 0.65–0.76) (12). Meanwhile, in England, girls in all the main minority ethnic groups are about 50% less likely to be vaccinated than their White British counterparts (13). In Île-de-France, the region around Paris, a two-fold variation in vaccine uptake among the eight Departments was observed, with the share of the population that were migrants significant in explaining the difference (14).

Relating to screening uptake, the most useful insights into inequality come from the European Health Interview Survey. As with vaccination, women born outside the EU are about 50% as likely to have been screened. There is also a steep educational gradient (odds ratio [OR] 0.60 for intermediate and 0.27 for low versus higher education), with those in the lowest income quintile having an OR of 0.60 compared with those in the highest. All of these findings were significant (15). Another study using these data highlighted the potential for health system factors to influence these inequalities, finding that differentials were greatly reduced in countries where the screening program was organized rather than *ad hoc*, and where access to healthcare was good (16).

To equitably reduce the burden of cervical cancer in Europe, and have a realistic hope of achieving the World Health Organization (WHO) 2030 target (17) of cervical cancer elimination, action is urgently needed to identify and respond to barriers to screening uptake affecting those underserved. This review was undertaken to inform the development of interventions to improve cervical cancer screening uptake among underserved women as part of the EU-funded CBIG-SCREEN project which involves working with these women to co-create solutions (18).

To inform these actions, we report the findings of a scoping review of the barriers and facilitators of access to CCS programs and the interventions taken to improve uptake among population groups at risk of being underserved in Europe. They are by virtue of a range of characteristics known to be associated with disadvantage, discrimination, and marginalization, identified through an iterative process of discussion within the research team. To facilitate the use of our findings to inform policy, we structure the different issues into the level of the health system at which they arise and can best be addressed (19) including the health system level (macro level), the screening program level (meso level) and the individual/community level (micro level).

## Methods

### Aims

Our first aim was to describe the range of barriers and facilitators to cervical screening reported in the published literature among groups underserved, or at risk of being underserved, by CCS programs or health systems more broadly. Our second aim was to describe interventions that had been developed to improve uptake. We report our findings using the Preferred Reporting Items for Systematic Reviews and Meta-Analyses (PRISMA) guidelines for scoping reviews.

### Scope

The criteria for inclusion are papers:

(i) identifying barriers, facilitators and interventions related to uptake of cervical cancer screening;
(ii) targeting underserved populations (see below);
(iii) conducted in Europe;
(iv) reporting primary data; and
(v) published since 2000.

These are reported using the PICOS framework in Table 1.

**Table 1:** Inclusion/exclusion criteria.

P: Population/setting	Inclusion: Studies presenting primary data on ‘underserved’ groups (or those at risk of being underserved). Studies set in European countries. Studies published since 2000. Exclusion: Studies not presenting primary data on ‘underserved’ groups (or those at risk of being underserved) (see text). Studies set outside Europe. Studies published before 2000.
I: Intervention/exposure	Inclusion: Barriers and facilitators identified by underserved women/women at risk of being underserved, pertaining to any aspect of the CCS secondary prevention continuum, extending until discharge or diagnosis. Barriers and facilitators identified by those other than the target population (e.g., healthcare providers). Interventions to improve participation across the whole CCS secondary prevention continuum among women considered to be underserved. Exclusion: Interventions for primary prevention (e.g., HPV vaccination). Interventions for tertiary prevention (e.g., treatment of cervical cancer). Barriers and facilitators related to primary or tertiary prevention.
C: Comparison/control	Inclusion: Comparisons with experience of general population. Exclusion: No comparison with general population.
O: Outcome	Inclusion: Studies reporting CCS participation (e.g., uptake, adherence, etc.) and studies reporting barriers to, and facilitators of, CCS participation or interventions to improve participation.
S: Study design(s)	Inclusion: Systematic and scoping reviews were not included. No further restrictions on study types.

We now consider each factor in turn. A barrier was defined as a factor that obstructs or prevents the uptake of CCS; a facilitator as a factor that supports or promotes uptake of screening; and an intervention was defined as an activity designed to increase screening uptake by members of the population groups that are the subject of the review.

Groups at risk of being underserved were identified in an iterative process of discussion within the research team, many of whom have extensive experience in designing, managing, or evaluating cervical screening programs. The discussion was informed by the concepts of vertical and horizontal equity, seeking groups that do not receive equal cervical screening when they have an equal need or those who do not receive a level of services corresponding to their specific need (20, 21). The groups included were restricted to those who may benefit from cervical screening, i.e., adults with a cervix (including women, trans men, non-binary people, and inter-sex people with a cervix). The discussion concluded that the most appropriate approach was one that combined generic terms, such disadvantage, stigmatization, and discrimination, with more specific terms such as sex work, incarceration, communicable diseases, substance misuse, trafficking and exploitation, and ethnicity. These were then operationalized using synonyms, stems, and Boolean operators in the search strategy (Appendix).

The rationale for limiting our review to Europe is that it will be used to develop interventions there. We recognize that there is an extensive literature on inequalities of access to care from the USA, some of which can offer insights, but much of which is not easily transferable given the very different health system and political contexts. This is less of a problem with literature from other parts of the world but, even then it would be necessary to assess each study to determine its applicability to the European context, especially as, in some cases, the inequalities that will exist in Australasia, Canada, and Latin America will be overlaid by the complex legacy of colonialism. Restricting our review to European studies also avoided the need to scrutinize a large volume of literature addressing issues such as the extremely sparse infrastructure in many low-income countries.

Studies were required to report primary data relevant to the issues set out above. Existing systematic reviews *per se* were not included, although if identified, reference lists were considered. Beyond this constraint, no studies were excluded based on study design.

Our search included studies published between 2000 – July 2021. This restriction to studies published since 2000 was applied due to shifts in social, legal and health system factors over time which are likely to have impacted experience of barriers and facilitators, such as the establishment of anti-discrimination legislation and health service digitalisation (22).

### Search strategy

Our detailed search strategy, which follows from the criteria listed above, is reported in Appendix A. In brief, four electronic databases were searched for relevant documents: Medline (Ovid), EMBASE (Ovid), Global Health (Ovid) and PsychINFO (APA/Ovid) from March to July 2021. Papers in any language were included, although all but one included after initial screening were in English. The full text of the remaining two, in Dutch and one in Estonian, were machine translated. This search was supplemented by a combination of hand searching of reference lists of relevant included studies and forward citation searching. The final search results were exported into EndNote, and duplicates were removed. The search strategy was developed in collaboration with an experienced librarian at the London School of Hygiene and Tropical Medicine and further refined through team discussion.

We extracted data from studies focused on barriers and facilitators covering country of study, the underserved group, and the nature of the barriers/facilitators. With the intervention studies, we extracted information on the nature of the underserved group, country, aim of the study, study design, intervention, and outcomes.

To increase consistency among reviewers (SB, VK, MM), each screened a sample of 95 papers, discussed the results, and amended the screening and data extraction document before beginning screening for this review. Screeners worked in pairs and sequentially evaluated the titles, abstracts and then full text of all publications identified by our searches for potentially relevant publications. Any disagreements on study selection were resolved by consensus and discussion with other reviewers if needed.

Data extraction, allocation to themes, and interpretation of the findings was undertaken by two reviewers (SR and RG) for papers relating to barriers and facilitators. For papers relating to interventions, data were extracted by three reviewers (SB, RL, MM), followed by deductive categorization into micro/meso/macro level phenomena by reviewers (SR and RG).

As this was a scoping review, with very heterogenous studies, a comprehensive critical appraisal of studies was not appropriate.

### Data synthesis and analysis

Unlike a systematic review, where it is important to exclude studies that fail to meet pre-specified quality criteria lest they distort a central measure, this review sought to identify factors that plausibly and convincingly acted as barriers and facilitators and interventions which drew attention to increasing uptake screening. Thus, the quality of the methodology and, therefore, the validity of the findings of individual papers were not assessed.

The barriers, facilitators and interventions acting at each level (macro-, meso-, micro-) were described along with the groups affected.

## Results

**Figure 1:**
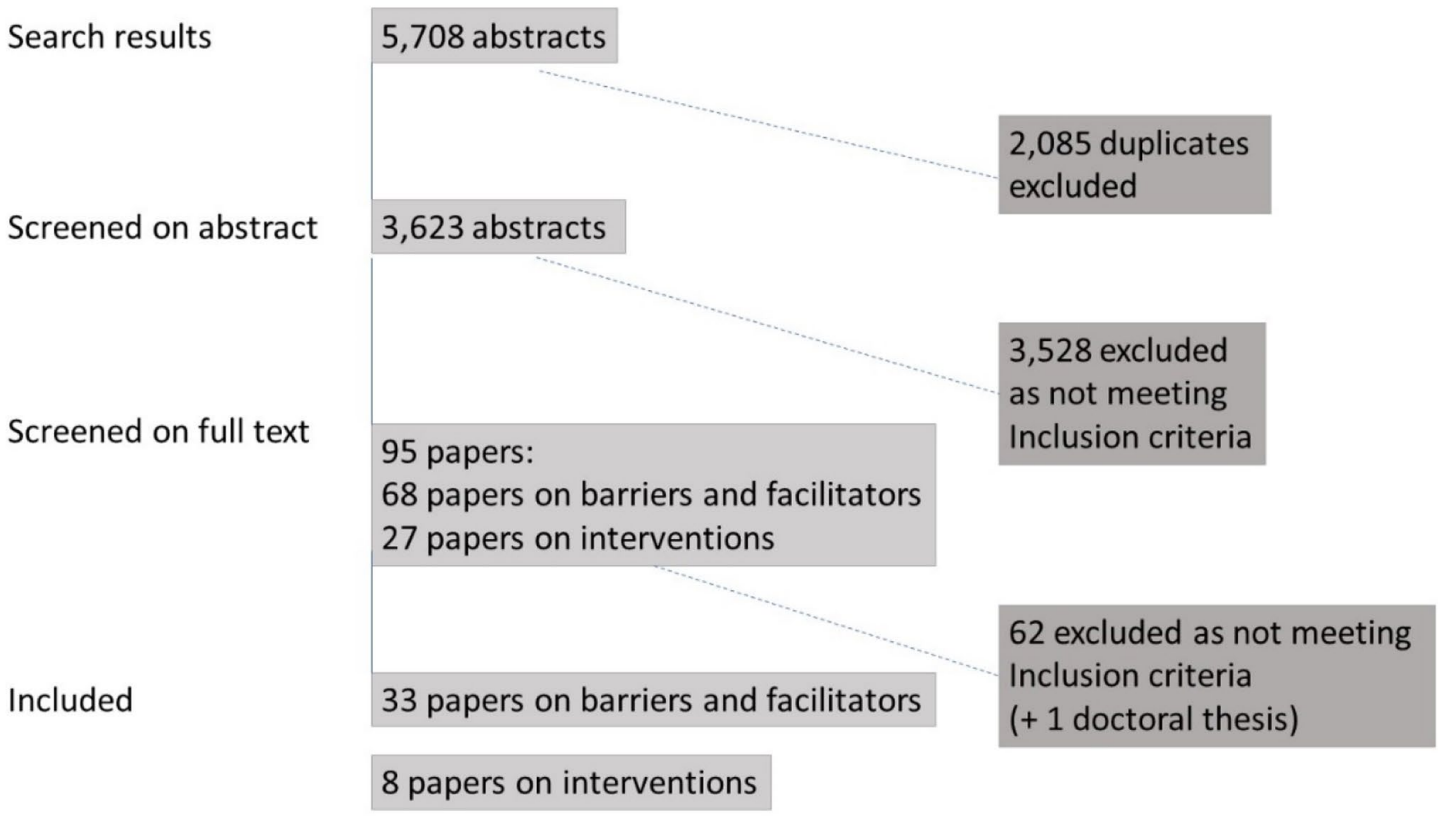
Study selection flow diagram.

After removing duplicates, 3,623 unique studies were identified. Screening of titles and abstracts reduced this to 95 studies, with 68 covering barriers/facilitators and 27 reporting interventions (Figure 1). 41 studies remained after full-text screening, with 33 related to barriers and facilitators (Table 2) and eight intervention studies (Table 3). Most studies were conducted in the UK (*n* = 16), with the remainder in Romania (*n* = 3), Denmark (*n* = 2), the Netherlands (*n* = 2), Norway (*n* = 2), Sweden (*n* = 2), Bulgaria (*n* = 1), Switzerland (*n* = 1), Estonia (*n* = 1), Finland (*n* = 1), France (*n* = 1), Poland (*n* = 1), and Portugal (*n* = 1). One study was conducted in two countries (Bulgaria and Romania).

**Table 2:** Overview of studies reporting barriers and facilitators within micro, meso and macro level framework.

No.	Reference—author(s) (year) [Country] underserved population	Barriers			Facilitators		
		Macro	Meso	Micro	Macro	Meso	Micro
1	Gele et al. (2017) (23) [Norway] Immigrant groups—Pakistani and Somali women	Lack of trust in health system; Inadequate access to primary care services of quality	Long waiting times; Lack of translated information provision in appropriate format; Lack of access to female clinicians	Limited cancer/screening awareness; Concern about stigma (sexual activity and FGM) Limited belief in the principle of prevention; Competing time and economic pressures		Improved dissemination of information; Improved access to female doctors; Institution of a recall system	
2	Azerkan et al. (2015) (24) [Sweden] Immigrant groups—Danish and Norwegian women	Lack of trust in health system	Complex care pathway; Impersonal correspondence; Previous negative experiences of care	Limited cancer/screening awareness; Previous trauma; Migration-related routine disruption; Limited belief in principle of prevention and state intervention			
3	Darwin and Campbell (2009) (25) [UK] Sexual minority women		Lack of inclusivity in campaign material	Concern about sigma due to sexual identity		Improved awareness training for staff	
4	Marlow et al. (2015) (26) [UK] Ethnic minority groups		Lack of suitable appointments	Limited cancer/screening awareness; Fear of results; Limited belief in the principle of prevention; Embarrassment			
5	Marques et al. (2021) (27) [Portugal] Migrant groups	Barriers registering for care services; Incorrect information held by registries	Lack of (translated) information; Complex care pathway; Lack of access to female / representative staff	Limited cancer/screening awareness; imited belief in the principle of prevention; Lack of health autonomy; Concern about stigma (FGM)	Continuity of care; Access to translation services	Access to self-sampling; Availability of time during appointments	
6	Edelman et al. (2013) (28) [UK] Women who self-identified as having a substance use problem and actively used in the past month			Concern about stigma (hygiene, drug use, sexual history) and triggering trauma; Fear of results; Competing time pressure (related to substance dependency); Low self-regard; Low engagement with principle of screening;			Option for family co-attendance
7	Salad et al. (2015) (29) [Netherlands] Ethnic minority group—Somali women		Lack of (translated) information; Lack of access to female staff	Limited cancer/screening awareness; Concern about stigma (FGM)			
8	Badre-Esfahani et al. (2021) (30) [Denmark] Ethnic minority groups – women from Middle Eastern and North African countries and Pakistan	Negative care experiences; Limited health system trust; Perception of hostility and structural racism	Limited access to female staff	Limited cancer/screening awareness; Embarrassment’ Concern about stigma (FGM); Limited belief in the principle of prevention; Fear of results; Competing time pressures		Provide targeted information and reminders; Increase routes of access to screening	
9	Tatari et al. (2020) (31) [Denmark] Ethnic minority and immigrant groups – women from Turkey, Iraq, Somalia, Lebanon, Syria, Saudi Arabia, Uzbekistan, Morocco, Pakistan, and Vietnam	Long waiting times; Mistrust of doctors	Lack of translated information	Limited cancer/screening awareness; Limited belief in the principle of prevention; Fear of results, stigma (FGM), pain and embarrassment.			
10	Akhagba (2017) (32) [Poland] Ethnicity minority /migrant group – Egyptian, Kenyan, Nigerian and Eritrean women	Long waiting times; Care costs	Lack of translated information; Lack of access to female staff	Limited cancer/screening awareness; Embarrassment; Lack of social network prompts			Increased social network support
11	Ekechi et al. (2014) (33) [UK] Ethnic minority groups – Black women predominantly from African or Caribbean backgrounds		Complex pathway	Limited cancer/screening awareness; Competing time pressures; Fear of screening test and results; Embarrassment	Improved education		
12	Condon et al. (2021) (34) [UK] Ethnic minority groups -Participants self-identified as Roma (from Slovakia and Romania) or as Gypsies, Travelers or Show people (described as Gypsy/Travelers)		Inaccessible language and lack of translated information; Complex pathways	Fear of results; Embarrassment			
13	Todorova et al. (2009) (35) [Bulgaria and Romania] representative sample of women from Bulgaria and Romania including ethnic minorities and immigrant women from Turkey and Hungary.	Long waiting times; Costs of care	Lack of reminders / prompts	Limited cancer/screening awareness: Lack of social network prompts; Competing time pressures			
14	Grandahl et al. (2012) (36) [Sweden] immigrant groups from Middle East, Africa, Asia, and East Europe		Lack of translated information; Lack of access to female staff	Deprioritisation of own health; Concern about stigma (sexual practices) and sampling process; Embarrassment; Lack of social network prompts	Increased healthcare accessibility; Improved experiences of healthcare		
15	Nelson et al. (2021) (37) [UK] Ethnic minority groups – South Asian, East European, Chinese, Black African and Caribbean women	Experiences of racism and discrimination; Incorrect patient contact data	Lack of translated information; Lack of access to female staff;	Limited cancer/screening awareness; Competing time pressures (work and care); Fear of pain; Concern about stigma (FGM); Embarrassment; Lack of social network prompts		Flexibility of appointments; Provision of prompts/ reminders	Increased prompts through social networks
16	Hamdiui et al. (2020) (38) [Netherlands] Migrant groups – Turkish and Moroccan women		Lack of (translated) information about screening and services; Lack of access to female staff	Limited cancer/screening awareness; Low engagement with principle of screening; Embarrassment; Limited social-network prompts; Fear of test, results and stigma (sexual activity); Competing pressures (work and care)		Reduced appointment length; Provision of information leaflets and reminders	Improved awareness of screening and cervical cancer; Improved social network support around screening
17	Andreassen et al. (2017) (39) [Romania] Ethnic minority group – Roma women	Costs of care; Perception of hostility and structural racism; Long waiting times	Lack of invitation from medical staff	Limited cancer/screening awareness; Unclear options for follow-up; Low engagement with principle of screening;		Increasing awareness of cervical cancer and screening	
18	Goutard et al. (2009) (40) [France] Women with physical disabilities	Physical access barriers to care settings				Screening enabled in multi-disciplinary settings	
19	Abdullahi et al. (2009) (41) [UK] Migrant groups – Somali women		Lack of translated information; Lack of access to female staff and convenient appointment times	Limited cancer/screening awareness; Embarrassment; Competing time pressures (care); Concern about stigma (FGM); Low engagement with principle of screening; Fear of screening test		Cultural sensitivity training for staff; Provision of prompts and translated information; Option for community co-attendance	
20	Jackowska et al. (2012) (42) [UK] Central and Eastern migrant groups – Polish, Slovak, and Romanian women	Health system distrust	Lack of translated information	Limited cancer/screening awareness; Migration-related time pressures/ routine disruption	Free access to care	Convenient appointment options with prompts / reminders;	
21	Møen et al. (2019) (43) [Norway] Immigrant groups		Complex care pathway; Lack of translated and culturally sensitive information provision; Lack of access to female staff	Limited cancer/screening awareness		Improved access to female staff	
22	Thomas et al. (2005) (44) [UK] Ethnic minority groups – African, Caribbean, Gujarati, Pakistani, Greek, and Arabic women	Adverse prior care experiences; Long waiting times; Health system distrust; Perception of hostility and structural racism	Lack of translated information and invitations to screening; Lack of cultural competence among staff	Limited cancer/screening awareness; Concerns about stigma		Provision of community education and mobile clinics; Improving access to female staff; Improved cultural awareness and access to translation services	
23	Cadman et al. (2012) (45) [UK] Women who have experienced sexual abuse		Lack of access to female staff; Limited trauma competence among staff	Challenges trusting healthcare staff; Concern about pain and emotional trauma;		Continuity of care and option for chaperone; Enabling autonomy over sample collection	
24	Marlow et al. (2015) (46) [UK] Older women (aged 50–65 years) from ethnic minority and lower socioeconomic groups		Challenge booking suitable appointment times	Limited cancer/screening awareness; Low engagement with principle of screening; Embarrassment			
25	Marlow et al. (2019) (47) [UK] Ethnic minority groups – women from Indian, Pakistani, Bangladeshi, Caribbean, African, Black British, Black Other, and White Other backgrounds		Unsuitable appointment times; Lack of invitations and reminders; Negative previous care experiences	Limited cancer/screening awareness; Low engagement with principle of screening; Lack of social network prompts; Fear of screening environment and process (hygiene, pain, issues related to FGM, and perceived risk of contracting cancer); Embarrassment; Competing time pressures			
26	Idehen et al. (2020) (48) [Finland] Migrant group - African women	Perception of hostility and structural racism	Negative previous experiences of screening; Lack of translated information	Limited cancer/screening awareness	Free access to care	Increased routes to access screening; Cultural competence among staff and in screening promotion; Provision of translated information/ reminder letters	
27	Patel et al. (2020) (49) [UK] Migrant group—Eastern European women	Health system distrust; Limited access to primary care services	Lack of translated information	Limited cancer/screening awareness			Improved awareness of cervical cancer
28	Berner et al. (2021) (50) [UK] Transgender men and non-binary people	Experience of stigma based on gender status	Lack of inclusive and targeted information	Concern about stigma (gender status), gender dysphoria, identity disclosure and procedural pain; Embarrassment; Competing time pressures		Access to specialist trans services; Provision of inclusive and targeted information; Access to self-sampling; Delivery of a call-recall system	
29	Andreassen et al. (2018) (51) [Romania] Ethnic minority group – Roma women	Costs of care	Distance to services	Limited cancer/screening awareness; Fear of results; Competing time pressures			
30	Conway et al. (2014) (52) [UK] Ethnic minority / migrant group—Chinese women	Health system distrust; Low levels of care registration	Lack of invitation to screening	Limited cancer/screening awareness			
31	Anderson et al. (2013) (53) [Estonia] Migrant women, those not speaking native language (Estonian) and women with low income.			Limited cancer/screening awareness			
32	Forrest et al. (2004) (54) [UK] Ethnic minority / Migrant groups—Indian, Pakistani, African-Caribbean and white British					Provision of Self-testing option	
33	Catarino et al. (2016) (55) [Switzerland]* Women from migrant communities as well as women who were unemployed, and uninsured.	Cost barriers		Limited cancer/screening awareness; Time barriers (work); Fear of test process, results and clinic attendance;			

**Table 3:** Overview of included intervention studies.

No.	Underserved group	Reference -author(s) and year	Country	Aim	Study design	Intervention	Outcomes
1	Pakistani and Somali immigrant women	Qureshi et al. (2021) (56)	Norway	To evaluate the effect of a community-based intervention aimed at increasing participation in the screening program among women from Pakistani and Somali groups.	Non-randomized controlled trial	The intervention was a presentation in Urdu and Somali on cervical cancer and CCS, including practical information, e.g., how to organize an appointment, with opportunity for questions. The control group did not receive this intervention.	Uptake of screening increased in the intervention group (46 to 51%) by significantly more than in the control group (44% to 45.5%), with an adjusted absolute difference in uptake of 0.03 (95% CI: 0.02–0.06). In subgroup analysis, absolute difference was significantly above zero for Somali women and first-generation migrants but not for Pakistani women and second-generation migrants.
2	Women with moderate and severe learning disabilities	Biswas et al. (2005) (57)	UK	To investigate whether one-to-one counseling can increase CCS uptake among women with moderate and severe learning disabilities.	Before-and-after study	One-to-one CCS counseling, using a toolkit containing visual education resources and guidance on consent, was delivered by specialist learning disability nurses.	Of the 160 women contacted to deliver counseling, nine (5.6%) were supported to complete CCS for the first time through the counseling process.
3	Women with low levels of education, migration women and older women	Radde et al. (2016) (58)	Germany	To investigate the effect of different models of invitation on CCS participation.	Randomized controlled trial	Participants received an invitation letter with a study ID card and pre-paid postage letter for correspondence (Arm A), the above + a brochure on cervical cancer (Arm B), or no invitation/brochure (Arm C).	Among those with low education (<12 years) the odds ratio of CCS participation in the intervention (Arm A + B) vs. control (Arm C) group was 2.86 (95% CI: 2.21–3.70). The equivalent figures for migrant women and women over 60 yrs. were 3.63 (1.89–6.96) and 2.45 (1.53–3.93) respectively.
4	Women from low-income groups	Sancho-Garnier et al. (2013) (59)	France	To compare participation and outcomes of CCS screening when women in low-income groups are offered HPV self-sampling vs. Pap-smear tests	Randomized controlled trial	Women were randomized to receive an invitation for a Pap-smear or an invitation for HPV self-sampling.	Uptake of screening was significantly higher among those invited for HPV self-sampling compared to those invited for Pap-smears (18.3% vs. 2.0% respectively, *p* < 0.001). However, a high proportion of both groups did not comply with follow-up in the instance of positive results (44 and 41% respectively)
5	Women from lower socioeconomic groups	Alfonzo et al. (2016) (60)	Sweden	To study the effect of abolishing fees for CCS in low resource areas already exposed to interventions to improve uptake	Randomized controlled trial	An invitation stating either that the test was offered for free (intervention group) or that it cost 100 SEK (control group).	Attendance did not differ significantly between women who were charged and those offered free screening (RR 0.93; CI 0.85–1.02).
6	Immigrant women	Møen et al. (2020) (61)	Norway	To determine whether CCS participation among immigrant women can be increased through multi-component interventions at general practitioner (GP) level	Cluster randomized controlled trial	Delivery of a CCS education session for GPs, a CCS mousepad for GPs and a CCS poster for waiting patients in GP practices to prompt CCS consideration.	After adjustment for baseline screening uptake levels, women in the intervention group had 1.24 times [95% CI, 1.11–1.38] higher odds of participating in screening than those in the control group. The effect was larger, although not significantly so, among those previously unscreened and those from Poland, Pakistan and Somalia.
7	Women in underserved communities (including: those unemployed, those with low levels of education and migrant communities)	Reques et al. (2021) (62)	France	To measure and compare the impact of different screening test offers on CCS uptake among underprivileged women in France.	Randomized controlled trial	All women were offered a reproductive health consultation, including information on screening. The experimental group were offered self-sample HPV testing, with referral if HPV results were non-negative, the control group were referred for Pap-smears	The hazard ratio (HR) for the screening test completion rate for the EG compared to the CG overall was 2.48 (95% CI:1.99–3.08) overall, the equivalent figures for those with no education/primary education only, those with irregular migration status, and those unemployed being 2.86 (95% CI:1.88–4.35), 2.56 (95% CI:2.00–3.26) and 2.94 (95% CI: 2.27–3.81) respectively.
8	Migrant women, women in low socioeconomic status groups and women who live in urban areas	De Nooijer et al. (2005) (63)	The Netherlands	Assess differences in CCS attendance according to source of invitation in groups with low CCS participation.	Retrospective cohort study	CCS invitations as part of population-based screening sent by either family doctors or the local public health department	Participation was 7.9% (95% CI: 7.5–8.3) higher among those invited by their family doctor vs. those invited by the local public health department. Among those born in Morocco, Turkey, Suriname and the Netherlands Antilles/Aruba the difference was 17.2% (95% CI: 15.2–19.2), among those affected by low socioeconomic status the difference was 11.6% (95% CI: 10.4–12.7) and among those living in the most urbanized areas the difference was 13.0% (95% CI: 12.3–13.6).

The population groups most commonly featuring in studies of barriers and facilitators were immigrant and migrant groups (*n* = 16) and ethnic minority women (*n* = 16). Less frequently featured populations included LGBTQI+ groups (*n* = 2), people who inject drugs (PWID) (*n* = 1), women with physical disabilities (*n* = 1), and women who have experienced sexual abuse (*n* = 1). Two studies addressed groups characterized by more than one factor. One study focused on women with lower school education, migrant women, and older women. Most studies focused on the perspectives of groups at risk of being underserved only (*n* = 26). Three studies included the perspectives of both healthcare professionals and underserved groups, and two included healthcare professionals or community workers only. Most studies focused on barriers rather than facilitators.

Interventions studied predominantly focused on psychological capability barriers (e.g., increasing understanding around cervical cancer and CCS) and physical opportunity barriers (e.g., sending invites, removing financial barriers, and offering HPV self-testing). Measures of effectiveness were mostly screening completion (recorded or self-reported) rather than treatment outcomes or survival rates. They comprised one non-randomized controlled trial, one before-and-and-after study, one retrospective cohort study and five randomized controlled trials.

The findings relating to barriers, facilitators, and interventions, presented according to the system level, are as follows.

### Macro level

There were many structural barriers to accessing any form of healthcare, with implications for screening, related to healthcare financing, health system bureaucracy, and trust in the health system.

Financial barriers, including lack of insurance coverage and concern about out-of-pocket costs, including ‘under the table’ costs, featured in four papers, all affecting ethnic minority groups in Central and Eastern Europe (32, 35, 39, 51). Two studies identified removal of financial barriers as a facilitator, in ethnic minority and migrant groups in the UK, and in Finland (42, 48).

Bureaucracy-related barriers were identified across four studies including migrant and ethnic minority women, three of which were based in the UK (27, 37, 49, 52). These barriers included challenges registering with primary care services (27, 49, 52), difficulties obtaining paperwork required to access services (27), and incorrect information on registers that prevented invitations being sent and engagement with screening services (27, 37).

A third barrier at this level was lack of trust. This was reported in studies of migrant women, those in ethnic minority groups and transgender men. The studies were from Norway, Sweden, Romania, and Finland, with two from Denmark, and four from the UK. Mistrust predominantly manifested as reputational concern about service quality or personal of experiences using the service, often involving discrimination (23, 24, 30, 31, 37, 39, 42, 44, 48–50, 52, 64). Conversely, previous positive health system experiences and perceptions of the system valuing women’s health were identified as facilitators in Sweden (36).

Only one intervention study was identified at the macro level. Contrary to reports (highlighted above) that removing cost barriers could facilitate access, this study, which randomized women living in a low-income urban area in Sweden to free screening or paying the standard fee (SEK 100 [€11]), found no significant difference in participation according to care fees (RR 0.93; CI 0.85–1.02) (60). However, the study design applied may have meant women did not realize the fee had been abolished for them. Additionally, as the authors noted, their finding conflicted with that from a Finnish study (65) on breast cancer screening finding that price was indeed a barrier to uptake.

### Meso level

At the screening service (meso) level, factors impacting engagement with screening were identified along the screening process. Five core themes were identified: information provision, prompts to participate in screening, screening pathway navigation, screening access options and staff interactions.

Nineteen studies described problems with information provided, including lack of cultural relevance or inclusivity, or failure to translate material (23–25, 27, 29, 31, 32, 34, 36–38, 41–45, 48–50, 63). While these problems affected many groups, migrant populations and those in ethnic and sexual minority groups appeared particularly affected. It follows that provision of targeted (30, 50) and accessible information (38) disseminated in appropriate formats and languages (23, 27, 41, 48) were highlighted as facilitators, with examples of good practice from Denmark, Finland, Romania, Sweden, Netherlands, Norway, and the UK.

A failure to provide prompts or invitations was a frequently cited barrier to participation (35, 37, 44, 47, 50, 52). In some cases this was due to inaccurate contact information held by screening services, an issue whose solution lies at the macro level where registry data are managed (37, 44). The use of call/recall systems (23, 50), reminder letters or phone calls (37, 38, 41, 42, 48) and proactive encouragement from healthcare professionals (41, 48) all acted as facilitators.

Those who decide to undergo screening may need help to navigate the system. This was often difficult, especially for migrant and ethnic minority groups across five studies in multiple country settings: Norway, Romania, and England. Specific navigation barriers included long waiting times for screening (23) and difficulty in booking suitable appointments (33, 34, 39, 41, 47). Additionally, complex pathway designs (24, 27, 33, 34, 63) created barriers for some migrant and ethnic minority groups (24, 27, 33, 34, 63). Distance to services was also a barrier facing Roma women in Romania (51).

Concerning routes of access, multiple studies identified screening care integration with other services (30, 40, 48), including enabling access through midwifery and multi-disciplinary care environments, as a facilitator of care.

Several studies identified flexibility in screening clinics as a facilitator, including extending appointment times and booking options (37, 42), providing mobile clinics (44), and enabling people within a community to attend together (41). The benefit of flexibility extended to how screening was undertaken, with options of chaperones, control over how clinicians perform the test, and self-testing all identified as facilitators (27, 45, 50, 54).

The final meso-level theme for barriers and facilitators related to interactions with screening staff. Several studies, not specific to any particular group or geographic area, highlighted uptake barriers relating to previous traumatic and negative care experiences (24, 28), embarrassment about the private nature of the examination (26, 30–34, 36–38, 41, 47, 49, 50) and perceptions of discrimination and stigma (23, 25, 27–30, 36–38, 41, 44, 50).

Some of these barriers also crossed to the micro level as they touched on issues such as access to female staff (44, 63), staff from similar backgrounds (48), and staff able to translate into the users’ languages (27, 41, 48). Some studies also highlighted how training could facilitate uptake by those identifying as trans or affected by female genital mutilation (25, 41, 44, 48, 50).

Interventions at the meso-level included information provision, prompts to participate in screening, choice of ways to access screening, and support to navigate the screening pathway.

#### Information provision and prompts

Two studies examined the effectiveness of invitations, with one simultaneously studying the impact of information provision. The latter was performed in Germany (58) and randomized participants to receive an invitation, an invitation with a brochure, or no invitation at all (control). The addition of the invitation increased uptake from 85% to 92%, but the further addition of the brochure had little effect. The invitation letters were most effective with women who had never been screened, with older women, those with low educational attainment and migrants.

The second study, based in the Netherlands (63), compared the impact of invitations from GPs with those from the local public health department. Invitations from GPs increased participation by 7.9% (95% CI: 7.5–8.3), and more so among migrants from Morocco, Turkey, Surinam, and the Netherlands Antilles/Aruba, and those on low incomes, in urban areas and in the lowest age group.

#### Accessible education materials

Only two studies were found that intervened to address cultural relevance, lack of inclusivity, or failure to translate material. One, from Norway (56), used a community-based intervention among women from Pakistani and Somali communities with practical information presented verbally in Urdu or Somali. Screening uptake was almost five percentage points higher in the intervention group (from 45.9% to 50.8%), which reduced to 3% (95% CI 2%–6%) after adjusting for confounders. This study benefitted from the ability to link the subjects to registry data rather than relying on self-report, which tends to overestimate screening and varies with ethnicity. One controversial limitation that was noted was the non-involvement of men, recognizing the role they play in women’s decisions in certain cultures, with the authors speculating that including them might have had additional benefits.

The second study, from the United Kingdom (57), assessed the incremental benefit of offering one-to-one counseling to women with moderate and severe learning disabilities by a learning disability nurse. This achieved a six-percent increase in uptake, from 16% to 22% among women attending screening for the first time. To deliver the intervention, nurses were given a health education pack with pictures adapted for women with varying levels of learning ability and a decision-making pathway (offering guidance on consent) to support nurses. Although multiple women were prompted to participate in screening through this program, the uptake achieved was still very low, which the authors attributed to a range of factors, including the low risk that these women faced because of low sexual activity and problems cooperating with the procedure, in some case because of their physical disabilities.

#### Multicomponent intervention: physician reminder and education

One cluster randomized controlled trial in Norway (61) evaluated a package of measures delivered in general practice aimed at increasing migrant women’s participation in CCS. It had three parts (1): a 10- to 15-min targeted educational session for healthcare providers held during the lunch break at the general practice (2), a mouse pad to remind GPs of the intervention in their day-to-day work, and (3) a poster for waiting rooms with the message, “You can prevent cervical cancer with a simple test. Make an appointment with your doctor today!” in Somali, Polish, English, and Urdu. The proportion of immigrant women screened increased by 2.6% in the intervention group versus 0.6% in the control group. After adjustment for screening status at baseline, women in the intervention group were more likely to have participated in CCS (OR, 1.24 [95% CI: 1.11–1.38]). This was unchanged after adjustment for women’s characteristics and was reduced, but still significant, after further adjustment for GP characteristics (OR, 1.19 [95% CI, 1.06–1.34]). In subgroup analyses, the intervention increased participation most by women who had not previously been screened at baseline (OR, 1.35 [95% CI: 1.16–1.56]) and those from Poland, Pakistan, and Somalia (OR, 1.74 [95% CI: 1.17–2.61]), after adjusting for baseline screening status.

#### Innovations in access and improved navigation

A study in France (62) evaluated the impact of offering self-sampling CCS tests in a community disadvantaged due to factors such as low-income, low levels of education, and experience of homelessness and sub-standard housing. All women eligible and included in the study were offered an individual sexual and reproductive health consultation covering HPV infection, cervical cancer, and gynecological care and screening access information. The intervention group were provided with self-sampling HPV test kits, while the control group were referred for Pap-smear tests. Those randomized to self-sampling could take their sample at the clinic or at home, receiving a referral if a non-negative result was returned. Those provided self-sampling tests had over twice the likelihood of completing screening tests compared to those in the control group (hazard ratio = 2.48 [95%CI: 1.99–3.08]).

A randomized trial in the south of France (59) was undertaken among non-attenders to a first invitation for a Pap-smear. This group was sent either a mailed invitation and kit for HPV self-sampling (HPVHR test) or a 2nd invitation (reminder) to Pap-smears. The outcomes were participation and detection of cervical dysplasia. Self-sampling markedly increased participation, from 2.0% with Pap-smears (198/9,901) versus 18.3% with HPVHR tests (1,613/8,829). The main limitation was that, despite increased screening uptake and intensive follow-up, most women with positive HPV screening tests did not accept the subsequent invitation to have a Pap-smear followed by a colposcopy examination if indicated.

#### Micro level

At the individual and community (micro) level, barriers and facilitators related to themes of a lack of awareness about screening, fear of screening, competing pressures, and attitudes to screening and preventive care in general.

Limited awareness of cervical cancer or CCS was a common theme, featuring in 22 papers, often attributed to the suboptimal provision of information, something that crosses the micro and meso levels.

Many barriers related to awareness involved a lack of understanding about cervical cancer and associated risk factors (24, 30, 32, 33, 38, 39, 41, 47–49), and a lack of awareness of cervical screening and available screening services (23, 26, 27, 29, 31, 32, 35, 37, 38, 41–44, 47–50, 52, 63). These affected many groups, in particular migrant and ethnic minority groups, older women, transgender men, people who inject drugs (PWID) and those in socioeconomically deprived groups.

Measures to increase awareness of cervical cancer were identified as facilitators in several studies among ethnic minority groups (38, 39, 49).

Fear of screening also affected several groups and was reported in 24 papers. It took several forms, including aversion to the physical process of sample collection, concern about social interactions around screening (including stigma and embarrassment), and fear of receiving an adverse screening result.

Fear of pain was the main barrier related to the process of collecting a sample, featuring in seven papers and affecting several groups (23, 31, 33, 37, 45, 47, 50). However, an additional concern, reported only by migrant women, was the potential for screening to affect their virginity, with the associated stigma (36).

Concerns around the social experience of screening were also common, with embarrassment and fear of stigma featuring in 18 papers (23, 25, 27–32, 34, 36–38, 41, 44, 46, 47, 49, 50). Sub-themes of perceived or experienced stigma or dysphoria related to history of female genital mutilation (23, 27, 29, 30, 37, 47), gender status (25, 50), substance misuse history and sexual history also featured (28). While some studies described concern about health professionals engaging in stigmatizing behavior, others highlighted concern that participation in screening was itself stigmatized as it implied prior sexual activity (23, 36, 44). Others expressed fear of recalling trauma during previous sexual abuse (24, 28, 45).

Fear of what screening might reveal featured in 10 studies from many countries and population groups (24, 26, 28, 30, 31, 33, 34, 38, 47, 51), highlighting the importance of considering downstream care and support and related perceptions.

Other micro themes included competing pressures, the impact of screening perceptions within social networks, and beliefs about screening and prevention in principle.

Many, from a wide array of groups, cited competing priorities as a barrier (23, 24, 26, 28, 30, 33, 35, 37, 38, 41, 42, 47, 50, 51). Childcare and work both featured as competing priorities in three studies involving women from ethnic minority groups (37, 38, 41). Some PWID described pressures arising from substance use (28). Some migrant groups faced difficulties associated with the complex challenges arising from the migration process (24, 42). Meanwhile, many simply deprioritized screening owing to feelings of low self-worth (28, 36, 45, 66).

Many women described how social networks could support attendance (28, 32, 37, 38) with one study finding that PWID may benefit from having an option to be joined by family members when attending screening (28). However, particularly in migrant and ethnic minority groups, social networks could be a barrier to participation, especially where discussion of sexual health was ‘taboo’ (27, 36–38, 47).

Concerning beliefs relating to screening and prevention, many, especially those in migrant and ethnic minority groups, held a fatalistic belief about cancer or disengaged from the idea of prevention, seeing screening results as something that should not be sought (23, 27, 30, 31, 38, 39, 41, 47).

We did not identify any interventions at the micro level.

## Discussion

A first step in understanding the reasons behind the mismatch between intentions and outcomes of health policy is to appreciate the reality faced by people who are constrained by poverty, geographical isolation, or other disadvantages.

This review shows how women, and those with cervixes, eligible for CCS face a wide array of barriers to participation, ranging from limited awareness of screening services to a lack of culturally acceptable services which can be afforded and easily reached without fear of stigma. Positively, there are many ways to facilitate uptake, including interventions with proven effectiveness. The latter reduce barriers to participation by providing information on what to do, especially when it is in a language that is understood and is culturally appropriate, reducing financial, temporal, and geographic barriers and by introducing easier ways to obtain samples, in particular self-sampling. Collectively, these findings offer many ideas that can help reduce the inequitable burden of cervical cancer in Europe.

### Implications for policy and practice

There are many reasons why disadvantaged women are at greater risk of both developing cervical cancer and experiencing its progression, too often to preventable death. These groups start at higher risk, particularly from infection with HPV and HIV or tobacco use, compounded by low uptake of HPV vaccination and screening. Most European countries have implemented organized cervical screening programs, either with Pap smear or HPV testing, although there is still much to do to implement solutions which address barriers across the care pathway. Underserved women find themselves having to overcome multiple barriers to obtain screening, from finding where the services are, to making an appointment, paying out-of-pocket expenses, and experiencing unwelcoming care settings. Some women are especially likely to face psychological and emotional barriers and report economic, social, and healthcare system barriers. For example, migrant women from countries where girls face barriers to education may find it difficult to navigate a foreign healthcare system. Few healthcare systems proactively seek to meet the needs of minorities, and although civil society organizations may compensate for this, there is only so much that they can do.

This review builds on previous literature highlighting the complex interplay of factors influencing the uptake of cervical screening in the general population (67–69). The studies we have reviewed point to some general points. There is a diversity of barriers and facilitators which require a range of interventions if they are to be overcome. Interventions evidenced within this scoping review addressed the need to: (i) increase access/participation, (ii) provide culturally appropriate information, and (iii) improve the experience of being screened.

It goes without saying that universal access to easily accessible screening services is important and seems intuitive, and consistent with economic theory, that removal of financial barriers would be important, so the findings of the Swedish study should be interpreted with caution and may not be generalisable to other settings where there are also organizational barriers, where the fee is higher, or where levels of income support are lower (24).

Screening should also be organized as a comprehensive system that goes all the way from maintaining accurate population registers to ensuring follow-up of those found to have positive results (67). Good communication between providers and service users is imperative. Invitations to screening need to be timely, culturally sensitive, and accessible (focusing on screening/cancer-specific information). Perhaps the best evidence relates to self-sampling for HPV, shown in many studies to be effective (59, 62, 70–72), especially for some populations disadvantaged by traditional programs. However, there is scarce evidence on how interventions can be integrated into existing healthcare systems or can enhance women’s ability to make informed decisions. It is clear from this review that diverse efforts sensitive to a wide array of barriers are needed to meet the WHO 2030 targets for eliminating cervical cancer as a public health problem and to reduce inequalities in its incidence and mortality.

### Strengths and limitations

The review is, to our knowledge, the first to examine the entire range of barriers and facilitators to participation in CCS and potential interventions to increase uptake across identifiable population groups in Europe, with a focus on those at risk of being underserved by CCS programs.

The review focused on Europe because it is feeding directly into an EU project (CBIG-SCREEN) but this clearly limits generalisability to populations in other parts of the world. Another potential limitation is the use of *a priori* definitions of underserved populations. This was necessary to develop a search strategy capable of identifying relevant literature. However, it risks inadvertently excluding studies on underserved groups not recognized as being so. It is important to acknowledge that, even when insights into the barriers and facilitators faced by certain groups are obtained, these are not necessarily generalizable to the same groups elsewhere. People are not defined by a single characteristic and many of those that disadvantage individuals interact, either to exacerbate or mitigate their situation, a phenomenon termed intersectionality. The challenge of generalisability is compounded by the imbalanced geographical distribution of studies, with most studies of migrant and ethnic minority women coming from the United Kingdom. Consequently, a cautious approach is required when applying insights obtained from this review to settings other than where the research was undertaken. To aid generalisability, funders should prioritize future research in this field in other settings, as well as including possibilities to probe intersectionality.

The use of macro, meso, and micro levels allowed us to take a structured approach to synthesis and analysis that aligned with wider approaches to cervical screening policy and interventions in Europe (i.e., through CBIG-SCREEN). However, the distinction between categories was occasionally blurred. Awareness is one such example, as it transcends micro and meso levels. Undeniably, providing information on services has a role in improving awareness of cervical screening and its indications; however, cervical screening services cannot be held solely accountable for low levels of health literacy.

As this is a scoping review, the quality of the papers was not appraised in detail. Additionally, the heterogeneity and sparsity of the intervention studies precluded a meta-analysis. We must also consider that none of the authors falls into any of the underserved groups, something that may, sub-consciously, have influenced our interpretation of the results, although care was taken to challenge positionality and address reflexivity during the analysis and write-up.

## Conclusion

Although screening is a highly effective means of preventing invasive cervical cancer, it remains inaccessible to many women in Europe, for a wide range of reasons. Yet there is much that can be done to change this situation by removing financial, cultural, and other barriers and by providing services that respond to the needs and expectations of those groups that are disadvantaged. This project, CBIG-SCREEN, is now taking forward the findings of this review to engage with these several of these groups in three European countries to co-create models of care that they will feel a sense of ownership over.

## Author contributions

RG led the review. SB initiated the review protocol. RG and SR revised the review protocol and search strategy, participated in the writing, reviewing and revising the final versions of the report and coordinated the process of getting it to publication standards. SB, RG, and SR conducted all the screening, extraction, and analysis of texts and created the tables and figures. RL acted as second screener. VK led the design and refined the initial search strategy and study protocol and was responsible for conducting the electronic database searches and removing duplicate articles. MM conceived and scoped the review, provided expert advice, made critical comments that helped the interpretation of results, and revised reviewed the data extraction sheet, reviewed, and revised the report. All authors contributed to the article and approved the submitted version.

## Funding

CBIG-SCREEN has received funding from the EU’s Horizon 2020 research and innovation program under grant agreement No 964049. The work was supported by European Commission’s Horizon 2020 program as part of the CBIG-SCREEN research project; however, the views expressed do not necessarily reflect the European Commission’s official policies. The funders had no role in study design, data collection and analysis, decision to publish, or preparation of the manuscript.

## Conflict of interest

The authors declare that the research was conducted in the absence of any commercial or financial relationships that could be construed as a potential conflict of interest.

## Publisher’s note

All claims expressed in this article are solely those of the authors and do not necessarily represent those of their affiliated organizations, or those of the publisher, the editors and the reviewers. Any product that may be evaluated in this article, or claim that may be made by its manufacturer, is not guaranteed or endorsed by the publisher.
