# Supplementary material for: Factors influencing the participation of groups identified as underserved in cervical cancer screening in Europe: a scoping review of the literature
Source: Front Public Health. 2023 May 25;11:1144674. doi: 10.3389/fpubh.2023.1144674 (PMC10247980; doi:10.3389/fpubh.2023.1144674)
Supplement: Supplementary file 1 [file Table_1.DOCX]

**Appendix: Search Strategy**

Proposed systematic review question:

What are the barriers to, and facilitators of, access to cervical cancer screening (CCS) experienced by underserved groups and what are the most effective ways to improve CCS participation in these groups?

Databases:

1. Medline (Ovid)
2. EMBASE (Ovid)
3. Global Health (Ovid)
4. PsychINFO (APA/Ovid)

Other methods:

- Handsearching of reference lists of relevant included studies
- Forward citation searching of relevant included studies

**Medline** was searched using the OvidSP interface <1946 to July 13, 2021>

1. (Vulnerab* or stigma* or Underprivilege* or Marginali#e* or Exclu* or "Hard-to-reach" or "Difficult to reach" or Disadvantage* or Underserved or Discriminat* or impoverished or Depriv* or Unemploy* or Poverty or Poor or Homeless* or "low income" or Uneducated or rural* or ((Soci* or economic*) adj3 (status or class or position or depriv*)) or ((Low or poor* or limited) adj3 educat*)).mp. [mp=title, abstract, original title, name of substance word, subject heading word, floating sub-heading word, keyword heading word, organism supplementary concept word, protocol supplementary concept word, rare disease supplementary concept word, unique identifier, synonyms] 2109327

2. (Disabilit* or disabled).mp. [mp=title, abstract, original title, name of substance word, subject heading word, floating sub-heading word, keyword heading word, organism supplementary concept word, protocol supplementary concept word, rare disease supplementary concept word, unique identifier, synonyms] 340582

3. (LGBT* or lesbian* or bisexual* or gay or homosexual*).mp. [mp=title, abstract, original title, name of substance word, subject heading word, floating sub-heading word, keyword heading word, organism supplementary concept word, protocol supplementary concept word, rare disease supplementary concept word, unique identifier, synonyms] 44831

4 "sexual minorit*".mp. [mp=title, abstract, original title, name of substance word, subject heading word, floating sub-heading word, keyword heading word, organism supplementary concept word, protocol supplementary concept word, rare disease supplementary concept word, unique identifier, synonyms] 3297

5 ("non-binary" or "gender non-conforming" or "gender fluid" or "gender queer" or genderqueer or transm#n or "trans m#n" or "trans male" or transmasculine or "trans masculine" or transmasc* or "cross dress*" or transgender or transsexual or transvestite* or FTM or "female to male").mp. [mp=title, abstract, original title, name of substance word, subject heading word, floating sub-heading word, keyword heading word, organism supplementary concept word, protocol supplementary concept word, rare disease supplementary concept word, unique identifier, synonyms] 38773

6 ((Sex* adj3 (work* or trade or industry or service*)) or Prostitut* or escort*).mp. [mp=title, abstract, original title, name of substance word, subject heading word, floating sub-heading word, keyword heading word, organism supplementary concept word, protocol supplementary concept word, rare disease supplementary concept word, unique identifier, synonyms] 19191

7 (Detained or Detention or Incarcerat* or Imprison* or prison* or jail* or inmate).mp. [mp=title, abstract, original title, name of substance word, subject heading word, floating sub-heading word, keyword heading word, organism supplementary concept word, protocol supplementary concept word, rare disease supplementary concept word, unique identifier, synonyms] 43509

8 (("human immunodeficiency virus" or HIV) adj3 (positive or living or infection*)).mp. [mp=title, abstract, original title, name of substance word, subject heading word, floating sub-heading word, keyword heading word, organism supplementary concept word, protocol supplementary concept word, rare disease supplementary concept word, unique identifier, synonyms] 263189

9 ((Hepatitis adj3 (B or C)) or Blood-borne virus*).mp. [mp=title, abstract, original title, name of substance word, subject heading word, floating sub-heading word, keyword heading word, organism supplementary concept word, protocol supplementary concept word, rare disease supplementary concept word, unique identifier, synonyms] 181482

10 ((Mental* or Psych*) adj3 (condition* or ill* or disorder* or issue* or diagnos* or problem*)).mp. [mp=title, abstract, original title, name of substance word, subject heading word, floating sub-heading word, keyword heading word, organism supplementary concept word, protocol supplementary concept word, rare disease supplementary concept word, unique identifier, synonyms] 425350

11 (((Substance or alcohol* or drug) adj3 (disorder* or use* or abuse* or misuse or addict* or depend* or problem* or issue*)) or Alcoholi*).mp. [mp=title, abstract, original title, name of substance word, subject heading word, floating sub-heading word, keyword heading word, organism supplementary concept word, protocol supplementary concept word, rare disease supplementary concept word, unique identifier, synonyms] 462903

12 (Sexual* adj3 (abuse* or assault* or trauma*)).mp. [mp=title, abstract, original title, name of substance word, subject heading word, floating sub-heading word, keyword heading word, organism supplementary concept word, protocol supplementary concept word, rare disease supplementary concept word, unique identifier, synonyms] 25814

13 (ethnic* or minorit* or BME or BAME or race or racial or caste or indian* or pakistani* or chinese or arab* or bangladeshi* or gypsy or gypsies or traveller* or asian* or black or african* or caribbean or "central and eastern european*" or "central european*" or "eastern european*" or iraqi* or somali* or bosnian* or serbian* or brazilian* or surinamese or "north african*" or "south american*" or romanian* or roma* or ex-yugoslavia or turkish or albanian* or russian*).mp. [mp=title, abstract, original title, name of substance word, subject heading word, floating sub-heading word, keyword heading word, organism supplementary concept word, protocol supplementary concept word, rare disease supplementary concept word, unique identifier, synonyms] 1347283

14 (traffick* or refugee* or immigra* or migra* or foreigner* or "asylum-seek*").mp. [mp=title, abstract, original title, name of substance word, subject heading word, floating sub-heading word, keyword heading word, organism supplementary concept word, protocol supplementary concept word, rare disease supplementary concept word, unique identifier, synonyms] 527609

15 ((Cervical or Pap or Papanicolaou or HPV or "high-risk HPV" or hrHPV or "Human Papillomavirus" or smear) adj5 (screen* or test* or smear)).mp. [mp=title, abstract, original title, name of substance word, subject heading word, floating sub-heading word, keyword heading word, organism supplementary concept word, protocol supplementary concept word, rare disease supplementary concept word, unique identifier, synonyms] 56185

16 1 or 2 or 3 or 4 or 5 or 6 or 7 or 8 or 9 or 10 or 11 or 12 or 13 or 14 4994768

17 europe/ or eastern europe/ or southern europe/ or western europe/ 112925

18 balkan peninsula/ or baltic states/ or vatican city state/ or Gibraltar/ 1370

19 (europe or balkan*or mediterranean or vatican or gibraltar).ti,ab. 116749

20 Andorra/ 32

21 Andorra.ti,ab. 67

22 Austria/ 20024

23 Austria.ti,ab. 13011

24 Baltic States/ 945

25 (Baltic adj1 (state? or countr* or region)).ti,ab. 626

26 exp Belgium/ 19619

27 Belgium.ti,ab. 16650

28 benelux/ 0

29 Benelux.ti,ab. 135

30 Croatia/ 7149

31 Croatia.ti,ab. 6546

32 Czech Republic/ 8087

33 Czechoslovakia/ 17905

34 (Czech Republic or Czechia or Czechoslovakia).ti,ab. 11694

35 Cyprus/ 1400

36 Cyprus.ti,ab. 2081

37 Denmark/ 51035

38 Denmark.ti,ab. 29069

39 Estonia/ 2500

40 Estonia.ti,ab. 2701

41 faroe islands/ 51035

42 Faroe Islands.ti,ab. 508

43 exp Finland/ 36755

44 Finland.ti,ab. 25692

45 exp France/ 104861

46 (France or French Republic).ti,ab. 65313

47 exp Germany/ 124196

48 Germany.ti,ab. 85148

49 Gibraltar/ 109

50 Gibraltar.ti,ab. 385

51 Greece/ 17800

52 (Greece or Hellenic Republic).ti,ab. 15803

53 Greenland/ 2511

54 Greenland.ti,ab. 3813

55 Hungary/ 18971

56 Hungary.ti,ab. 10919

57 Iceland/ 4642

58 Iceland.ti,ab. 4973

59 Ireland/ 18913

60 ((Ireland or Eire) not Northern Ireland).ti,ab. 17521

61 exp Italy/ 103080

62 (Italy or Italian Republic).ti,ab. 70128

63 Latvia/ 1329

64 Latvia.ti,ab. 1471

65 Liechtenstein/ 44

66 Liechtenstein.ti,ab. 325

67 Lithuania/ 2994

68 Lithuania.ti,ab. 2871

69 Luxembourg/ 731

70 Luxembourg.ti,ab. 1106

71 Malta/ 792

72 Malta.ti,ab. 1412

73 monaco/ 91

74 Monaco.ti,ab. 407

75 Netherlands/ 70716

76 (Netherlands or Holland).ti,ab. 52938

77 exp Norway/ 40766

78 Norway.ti,ab. 31986

79 Poland/ 50063

80 Poland.ti,ab. 25834

81 exp Portugal/ 14068

82 (Portugal or Portuguese Republic).ti,ab. 12930

83 San Marino/ 75

84 San Marino.ti,ab. 99

85 Scandinavia/ 5306

86 (((Scandinavian or Nordic) adj1 (countr* or state? or region)) or Scandinavia).ti,ab. 6284

87 Slovakia/ 3134

88 (Slovakia or Slovak Republic).ti,ab. 4367

89 Slovenia/ 2840

90 Slovenia.ti,ab. 3626

91 exp Spain/ 82103

92 (Spain or Balearic Islands or Canary Islands).ti,ab. 62069

93 Sweden/ 77278

94 Sweden.ti,ab. 48322

95 Switzerland/ 37089

96 (Switzerland or Swiss Confederation).ti,ab. 25422

97 exp United Kingdom/ or channel islands/ or "isle of man"/ 376540

98 (United Kingdom or UK or Great Britain or GBR or Northern Ireland or Scotland or Channel Islands or Isle of Man or (Wales not New South Wales) or (England not New England)).ti,ab. 218308

99 Moldova/ 715

100 Moldova.ti,ab. 564

101 exp Ukraine/ 16270

102 Ukraine.ti,ab. 5122

103 Albania/ 900

104 Albania.ti,ab. 1141

105 Armenia/ 1470

106 Armenia.ti,ab. 1176

107 exp Azerbaijan/ 1246

108 Azerbaijan.ti,ab. 1480

109 Belarus/ 2108

110 (Belarus or Byelarus or Byelorussia or Belorussia).ti,ab. 1631

111 exp "Bosnia and Herzegovina"/ 2220

112 (Bosnia or Herzegovina).ti,ab. 2469

113 Bulgaria/ 6474

114 Bulgaria.ti,ab. 4408

115 exp "Georgia (republic)"/ 1878

116 Georgia.ti,ab. not "georgia (u.s.)"/ 10458

117 Kosovo/ 239

118 Kosovo.ti,ab. 972

119 "Montenegro (republic)"/ 0

120 Montenegro.ti,ab. 888

121 "republic of north macedonia"/ 596

122 North Macedonia.ti,ab. 102

123 Romania/ 10417

124 Romania.ti,ab. 6116

125 exp Russian Federation/ 0

126 ussr/ 42807

127 (Russia or Russian Federation or USSR or Union of Soviet Socialist Republics or Soviet Union).ti,ab. 29818

128 exp Serbia/ 3364

129 Serbia.ti,ab. 4677

130 "Turkey (republic)"/ 0

131 (Turkey.ti,ab. not "Turkey (bird)"/) or (Anatolia or Asia Minor).ti,ab. 38794

132 or/17-131 [ALL COUNTRIES IN EUROPE] 1868551

133 *Vulnerable Populations/ 5279

134 *Minority Groups/ 8455

135 *Medically Underserved Area/ 3450

136 exp *Ethnic Groups/ 91503

137 *"Transients and Migrants"/ 9045

138 *Refugees/ or exp *Human Trafficking/ 9623

139 *Poverty Areas/ or *Poverty/ 19747

140 *Homeless Persons/ 6596

141 *Unemployment/ 3670

142 exp *Socioeconomic Factors/ 171934

143 *Rural Population/ 21286

144 exp *Disabled Persons/ 52482

145 *Homosexuality, Female/ or *Transsexualism/ or *Bisexuality/ or exp *"Sexual and Gender Minorities"/ or *Homosexuality/ or *Transgender Persons/ 20572

146 exp *Sex Workers/ 1987

147 *Prisoners/ 13471

148 *Hepatitis C/ or *Hepatitis B/ or *HIV Infections/ 230221

149 exp *Mental Disorders/ 1095036

150 exp *Substance-Related Disorders/ 221197

151 *Alcoholism/ 56541

152 exp *Sex Offenses/ 19160

153 *Uterine Cervical Neoplasms/pc [Prevention & Control] 5797

154 *Mass Screening/ or *"Early Detection of Cancer"/ 70302

155 *Cervical Intraepithelial Neoplasia/di, pc [Diagnosis, Prevention & Control] 2380

156 153 or 155 7779

157 154 and 156 2513

158 *papanicolaou test/ or *vaginal smears/ 11012

159 157 or 158 12851

160 15 or 159 60754

161 133 or 134 or 135 or 136 or 137 or 138 or 139 or 140 or 141 or 142 or 143 or 144 or 145 or 146 or 147 or 148 or 149 or 150 or 151 or 152 1682582

162 16 or 161 5601545

163 132 and 160 and 162 1853

164 limit 163 to (humans and yr="2000 -Current") 1380

**Embase Classic** was searched using Embase interface <1947 to 2021 July 14>

1 exp *vulnerable population/ 3795

2 exp *social exclusion/ 651

3 exp *ethnic group/ 39275

4 exp *poverty/ 13938

5 exp *social status/ 44355

6 exp *economic status/ or exp *unemployment/ or *employment status/ 34465

7 exp *homeless person/ 1161

8 exp *rural population/ 13920

9 *social stigma/ 3747

10 exp *social discrimination/ 8854

11 exp *educational status/ 9564

12 *disabled person/ or *mentally disabled person/ or *physically disabled person/ 20429

13 *lgbt people/ or *bisexual female/ or *homosexual female/ or *transgender/ 4593

14 *transgender/ or *female to male transgender/ 3947

15 exp *lgbtqia+ people/ or *"sexual and gender minority"/ or *asexual people/ or *intersex/ 8607

16 exp *sex worker/ 1063

17 *prisoner/ 9483

18 *Human immunodeficiency virus/ 54381

19 exp *hepatitis C/ or exp *hepatitis B/ 125693

20 exp *mental disease/ 1510957

21 exp *alcoholism/ or exp *substance abuse/ or exp *addiction/ 217088

22 exp *sexual abuse/ 10437

23 exp *migrant/ 18145

24 exp *asylum seeker/ or exp *refugee/ or exp *human trafficking/ 8650

25 1 or 2 or 3 or 4 or 5 or 6 or 7 or 8 or 9 or 10 or 11 or 12 or 13 or 14 or 15 or 16 or 17 or 18 or 19 or 20 or 21 or 22 or 23 or 24 1896724

26 *mass screening/ or *cancer screening/ 56999

27 *uterine cervix carcinoma in situ/ 8768

28 26 and 27 471

29 exp *papanicolaou test/ 4295

30 *vagina smear/ 4428

31 28 or 29 or 30 9056

32 (Vulnerab* or stigma* or Underprivilege* or Marginali#e* or Exclu* or "Hard-to-reach" or "Difficult to reach" or Disadvantage* or Underserved or Discriminat* or impoverished or Depriv* or Unemploy* or Poverty or Poor or Homeless* or "low income" or Uneducated or rural* or ((Soci* or economic*) adj3 (status or class or position or depriv*)) or ((Low or poor* or limited) adj3 educat*)).mp. [mp=title, abstract, heading word, drug trade name, original title, device manufacturer, drug manufacturer, device trade name, keyword, floating subheading word, candidate term word] 3075698

33 (Disabilit* or disabled).mp. [mp=title, abstract, heading word, drug trade name, original title, device manufacturer, drug manufacturer, device trade name, keyword, floating subheading word, candidate term word] 414562

34 (LGBT* or lesbian* or bisexual* or gay or homosexual*).mp. [mp=title, abstract, heading word, drug trade name, original title, device manufacturer, drug manufacturer, device trade name, keyword, floating subheading word, candidate term word] 49379

35 "sexual minorit*".mp. [mp=title, abstract, heading word, drug trade name, original title, device manufacturer, drug manufacturer, device trade name, keyword, floating subheading word, candidate term word] 4064

36 ("non-binary" or "gender non-conforming" or "gender fluid" or "gender queer" or genderqueer or transm#n or "trans m#n" or "trans male" or transmasculine or "trans masculine" or transmasc* or "cross dress*" or transgender or transsexual or transvestite* or FTM or "female to male").mp. [mp=title, abstract, heading word, drug trade name, original title, device manufacturer, drug manufacturer, device trade name, keyword, floating subheading word, candidate term word] 60582

37 ((Sex* adj3 (work* or trade or industry or service*)) or Prostitut* or escort*).mp. [mp=title, abstract, heading word, drug trade name, original title, device manufacturer, drug manufacturer, device trade name, keyword, floating subheading word, candidate term word] 24930

38 (Detained or Detention or Incarcerat* or Imprison* or prison* or jail* or inmate).mp. [mp=title, abstract, heading word, drug trade name, original title, device manufacturer, drug manufacturer, device trade name, keyword, floating subheading word, candidate term word] 57001

39 (("human immunodeficiency virus" or HIV) adj3 (positive or living or infection*)).mp. [mp=title, abstract, heading word, drug trade name, original title, device manufacturer, drug manufacturer, device trade name, keyword, floating subheading word, candidate term word] 359140

40 ((Hepatitis adj3 (B or C)) or Blood-borne virus*).mp. [mp=title, abstract, heading word, drug trade name, original title, device manufacturer, drug manufacturer, device trade name, keyword, floating subheading word, candidate term word] 297431

41 ((Mental* or Psych*) adj3 (condition* or ill* or disorder* or issue* or diagnos* or problem*)).mp. [mp=title, abstract, heading word, drug trade name, original title, device manufacturer, drug manufacturer, device trade name, keyword, floating subheading word, candidate term word] 393609

42 (((Substance or alcohol* or drug) adj3 (disorder* or use* or abuse* or misuse or addict* or depend* or problem* or issue*)) or Alcoholi*).mp. [mp=title, abstract, heading word, drug trade name, original title, device manufacturer, drug manufacturer, device trade name, keyword, floating subheading word, candidate term word] 739597

43 (Sexual* adj3 (abuse* or assault* or trauma*)).mp. [mp=title, abstract, heading word, drug trade name, original title, device manufacturer, drug manufacturer, device trade name, keyword, floating subheading word, candidate term word] 39036

44 (ethnic* or minorit* or BME or BAME or race or racial or caste or indian* or pakistani* or chinese or arab* or bangladeshi* or gypsy or gypsies or traveller* or asian* or black or african* or caribbean or "central and eastern european*" or "central european*" or "eastern european*" or iraqi* or somali* or bosnian* or serbian* or brazilian* or surinamese or "north african*" or "south american*" or romanian* or roma* or ex-yugoslavia or turkish or albanian* or russian*).mp. [mp=title, abstract, heading word, drug trade name, original title, device manufacturer, drug manufacturer, device trade name, keyword, floating subheading word, candidate term word] 1905079

45 (traffick* or refugee* or immigra* or migra* or foreigner* or "asylum-seek*").mp. [mp=title, abstract, heading word, drug trade name, original title, device manufacturer, drug manufacturer, device trade name, keyword, floating subheading word, candidate term word] 738897

46 32 or 33 or 34 or 35 or 36 or 37 or 38 or 39 or 40 or 41 or 42 or 43 or 44 or 45 6943394

47 ((Cervical or Pap or Papanicolaou or HPV or "high-risk HPV" or hrHPV or "Human Papillomavirus" or smear) adj5 (screen* or test* or smear)).mp. [mp=title, abstract, heading word, drug trade name, original title, device manufacturer, drug manufacturer, device trade name, keyword, floating subheading word, candidate term word] 125992

48 europe/ or eastern europe/ or southern europe/ or western europe/ 170495

49 balkan peninsula/ or baltic states/ or vatican city state/ or Gibraltar/ 1394

50 (europe or balkan*or mediterranean or vatican or gibraltar).ti,ab. 166033

51 Andorra/ 62

52 Andorra.ti,ab. 81

53 Austria/ 25606

54 Austria.ti,ab. 20422

55 Baltic States/ 754

56 (Baltic adj1 (state? or countr* or region)).ti,ab. 763

57 exp Belgium/ 28684

58 Belgium.ti,ab. 24940

59 benelux/ 90

60 Benelux.ti,ab. 182

61 Croatia/ 10718

62 Croatia.ti,ab. 10261

63 Czech Republic/ 14833

64 Czechoslovakia/ 16969

65 (Czech Republic or Czechia or Czechoslovakia).ti,ab. 18641

66 Cyprus/ 2682

67 Cyprus.ti,ab. 2897

68 Denmark/ 55966

69 Denmark.ti,ab. 39673

70 Estonia/ 4271

71 Estonia.ti,ab. 3800

72 faroe islands/ 344

73 Faroe Islands.ti,ab. 619

74 exp Finland/ 40954

75 Finland.ti,ab. 32806

76 exp France/ 139704

77 (France or French Republic).ti,ab. 102892

78 exp Germany/ 210854

79 Germany.ti,ab. 135530

80 Gibraltar/ 179

81 Gibraltar.ti,ab. 393

82 Greece/ 29953

83 (Greece or Hellenic Republic).ti,ab. 22431

84 Greenland/ 3405

85 Greenland.ti,ab. 4121

86 Hungary/ 23795

87 Hungary.ti,ab. 18310

88 Iceland/ 6693

89 Iceland.ti,ab. 6089

90 Ireland/ 37835

91 ((Ireland or Eire) not Northern Ireland).ti,ab. 157749

92 exp Italy/ 130324

93 (Italy or Italian Republic).ti,ab. 97774

94 Latvia/ 2688

95 Latvia.ti,ab. 2328

96 Liechtenstein/ 150

97 Liechtenstein.ti,ab. 380

98 Lithuania/ 4766

99 Lithuania.ti,ab. 3960

100 Luxembourg/ 1537

101 Luxembourg.ti,ab. 1458

102 Malta/ 1935

103 Malta.ti,ab. 2250

104 monaco/ 874

105 Monaco.ti,ab. 1193

106 Netherlands/ 89259

107 (Netherlands or Holland).ti,ab. 77585

108 exp Norway/ 47251

109 Norway.ti,ab. 40705

110 Poland/ 58165

111 Poland.ti,ab. 36665

112 exp Portugal/ 21960

113 (Portugal or Portuguese Republic).ti,ab. 19430

114 San Marino/ 124

115 San Marino.ti,ab. 134

116 Scandinavia/ 6065

117 (((Scandinavian or Nordic) adj1 (countr* or state? or region)) or Scandinavia).ti,ab. 8208

118 Slovakia/ 6181

119 (Slovakia or Slovak Republic).ti,ab. 6680

120 Slovenia/ 5592

121 Slovenia.ti,ab. 5676

122 exp Spain/ 105020

123 (Spain or Balearic Islands or Canary Islands).ti,ab. 84602

124 Sweden/ 88763

125 Sweden.ti,ab. 65327

126 Switzerland/ 46046

127 (Switzerland or Swiss Confederation).ti,ab. 50236

128 exp United Kingdom/ or channel islands/ or "isle of man"/ 464270

129 (United Kingdom or UK or Great Britain or GBR or Northern Ireland or Scotland or Channel Islands or Isle of Man or (Wales not New South Wales) or (England not New England)).ti,ab. 382352

130 Moldova/ 1313

131 Moldova.ti,ab. 1035

132 exp Ukraine/ 17544

133 Ukraine.ti,ab. 7664

134 Albania/ 1987

135 Albania.ti,ab. 1821

136 Armenia/ 2210

137 Armenia.ti,ab. 1788

138 exp Azerbaijan/ 2008

139 Azerbaijan.ti,ab. 2178

140 Belarus/ 3011

141 (Belarus or Byelarus or Byelorussia or Belorussia).ti,ab. 2623

142 exp "Bosnia and Herzegovina"/ 2954

143 (Bosnia or Herzegovina).ti,ab. 3189

144 Bulgaria/ 10304

145 Bulgaria.ti,ab. 7307

146 exp "Georgia (republic)"/ 2143

147 Georgia.ti,ab. not "georgia (u.s.)"/ 12346

148 Kosovo/ 602

149 Kosovo.ti,ab. 1338

150 "Montenegro (republic)"/ 807

151 Montenegro.ti,ab. 1242

152 "republic of north macedonia"/ 84

153 North Macedonia.ti,ab. 126

154 Romania/ 15587

155 Romania.ti,ab. 10359

156 exp Russian Federation/ 67350

157 ussr/ 48197

158 (Russia or Russian Federation or USSR or Union of Soviet Socialist Republics or Soviet Union).ti,ab. 40564

159 exp Serbia/ 6356

160 Serbia.ti,ab. 7392

161 "Turkey (republic)"/ 39529

162 (Turkey.ti,ab. not "Turkey (bird)"/) or (Anatolia or Asia Minor).ti,ab. 47271

163 or/48-162 [ALL COUNTRIES IN EUROPE] 2478464

164 25 or 46 7872257

165 31 or 47 126045

166 163 and 164 and 165 3618

167 limit 166 to (human and yr="2000 -Current") 2911

**Global Health** was searched using the OvidSP interface <1910 to July 28 2021>

1 exp risk groups/ 44025

2 exp socioeconomic status/ 36617

3 exp economically disadvantaged/ or unemployment/ 5271

4 rural communities/ 5276

5 deprivation/ or basic needs/ or living conditions/ or poverty/ 21548

6 exp homeless people/ 2693

7 exp people with disabilities/ 5663

8 exp people with mental disabilities/ or exp people with physical disabilities/ 3483

9 exp homosexual women/ 255

10 exp social stigma/ 5104

11 bisexuality/ or sexual behaviour/ or bisexual women/ 25663

12 exp sex workers/ 5223

13 exp prisoners/ 4587

14 exp human immunodeficiency viruses/ 187638

15 exp hepatitis b/ or exp hepatitis c/ 63340

16 exp mental disorders/ 84070

17 exp substance abuse/ 28901

18 exp alcoholism/ 11287

19 exp sexual abuse/ or exp sexual assault/ 4133

20 exp ethnic groups/ 80120

21 minorities/ or ethnic groups/ or groups/ or religion/ 60172

22 exp migrants/ 4469

23 exp refugees/ 4589

24 1 or 2 or 3 or 4 or 5 or 6 or 7 or 8 or 9 or 10 or 11 or 12 or 13 or 14 or 15 or 16 or 17 or 18 or 19 or 20 or 21 or 22 or 23 498127

25 exp cervical intraepithelial neoplasia/ 1986

26 exp papanicolaou testing/ 1047

27 screening/ 74905

28 (25 and 27) or 26 1571

29 (Vulnerab* or stigma* or Underprivilege* or Marginali#e* or Exclu* or "Hard-to-reach" or "Difficult to reach" or Disadvantage* or Underserved or Discriminat* or impoverished or Depriv* or Unemploy* or Poverty or Poor or Homeless* or "low income" or Uneducated or rural* or ((Soci* or economic*) adj3 (status or class or position or depriv*)) or ((Low or poor* or limited) adj3 educat*)).mp. [mp=abstract, title, original title, broad terms, heading words, identifiers, cabicodes] 477820

30 (Disabilit* or disabled).mp. [mp=abstract, title, original title, broad terms, heading words, identifiers, cabicodes] 27635

31 (LGBT* or lesbian* or bisexual* or gay or homosexual*).mp. [mp=abstract, title, original title, broad terms, heading words, identifiers, cabicodes] 16723

32 "sexual minorit*".mp. [mp=abstract, title, original title, broad terms, heading words, identifiers, cabicodes] 1101

33 ("non-binary" or "gender non-conforming" or "gender fluid" or "gender queer" or genderqueer or transm#n or "trans m#n" or "trans male" or transmasculine or "trans masculine" or transmasc* or "cross dress*" or transgender or transsexual or transvestite* or FTM or "female to male").mp. [mp=abstract, title, original title, broad terms, heading words, identifiers, cabicodes] 7344

34 ((Sex* adj3 (work* or trade or industry or service*)) or Prostitut* or escort*).mp. [mp=abstract, title, original title, broad terms, heading words, identifiers, cabicodes] 9286

35 (Detained or Detention or Incarcerat* or Imprison* or prison* or jail* or inmate).mp. [mp=abstract, title, original title, broad terms, heading words, identifiers, cabicodes] 9578

36 (("human immunodeficiency virus" or HIV) adj3 (positive or living or infection*)).mp. [mp=abstract, title, original title, broad terms, heading words, identifiers, cabicodes] 164759

37 ((Hepatitis adj3 (B or C)) or Blood-borne virus*).mp. [mp=abstract, title, original title, broad terms, heading words, identifiers, cabicodes] 72121

38 ((Mental* or Psych*) adj3 (condition* or ill* or disorder* or issue* or diagnos* or problem*)).mp. [mp=abstract, title, original title, broad terms, heading words, identifiers, cabicodes] 65428

39 (((Substance or alcohol* or drug) adj3 (disorder* or use* or abuse* or misuse or addict* or depend* or problem* or issue*)) or Alcoholi*).mp. [mp=abstract, title, original title, broad terms, heading words, identifiers, cabicodes] 112281

40 (Sexual* adj3 (abuse* or assault* or trauma*)).mp. [mp=abstract, title, original title, broad terms, heading words, identifiers, cabicodes] 5351

41 (ethnic* or minorit* or BME or BAME or race or racial or caste or indian* or pakistani* or chinese or arab* or bangladeshi* or gypsy or gypsies or traveller* or asian* or black or african* or caribbean or "central and eastern european*" or "central european*" or "eastern european*" or iraqi* or somali* or bosnian* or serbian* or brazilian* or surinamese or "north african*" or "south american*" or romanian* or roma* or ex-yugoslavia or turkish or albanian* or russian*).mp. [mp=abstract, title, original title, broad terms, heading words, identifiers, cabicodes] 504175

42 (traffick* or refugee* or immigra* or migra* or foreigner* or "asylum-seek*").mp. [mp=abstract, title, original title, broad terms, heading words, identifiers, cabicodes] 72294

43 29 or 30 or 31 or 32 or 33 or 34 or 35 or 36 or 37 or 38 or 39 or 40 or 41 or 42 1228868

44 ((Cervical or Pap or Papanicolaou or HPV or "high-risk HPV" or hrHPV or "Human Papillomavirus" or smear) adj5 (screen* or test* or smear)).mp. [mp=abstract, title, original title, broad terms, heading words, identifiers, cabicodes] 26669

45 europe/ or eastern europe/ or southern europe/ or western europe/ 535103

46 balkan peninsula/ or baltic states/ or vatican city state/ or Gibraltar/ 4911

47 (europe or balkan*or mediterranean or vatican or gibraltar).ti,ab. 44054

48 Andorra/ 20

49 Andorra.ti,ab. 20

50 Austria/ 8577

51 Austria.ti,ab. 5310

52 Baltic States/ 4836

53 (Baltic adj1 (state? or countr* or region)).ti,ab. 310

54 exp Belgium/ 10702

55 Belgium.ti,ab. 7247

56 benelux/ 40000

57 Benelux.ti,ab. 34

58 Croatia/ 5125

59 Croatia.ti,ab. 3369

60 Czech Republic/ 6249

61 Czechoslovakia/ 5002

62 (Czech Republic or Czechia or Czechoslovakia).ti,ab. 7171

63 Cyprus/ 1372

64 Cyprus.ti,ab. 1352

65 Denmark/ 18655

66 Denmark.ti,ab. 10991

67 Estonia/ 1848

68 Estonia.ti,ab. 1458

69 faroe islands/ 228

70 Faroe Islands.ti,ab. 229

71 exp Finland/ 15362

72 Finland.ti,ab. 9868

73 exp France/ 49786

74 (France or French Republic).ti,ab. 33686

75 exp Germany/ 52609

76 Germany.ti,ab. 30410

77 Gibraltar/ 65

78 Gibraltar.ti,ab. 76

79 Greece/ 12940

80 (Greece or Hellenic Republic).ti,ab. 8519

81 Greenland/ 826

82 Greenland.ti,ab. 919

83 Hungary/ 7582

84 Hungary.ti,ab. 5425

85 Iceland/ 1871

86 Iceland.ti,ab. 1637

87 ((Ireland or Eire) not Northern Ireland).ti,ab. 4470

88 exp Italy/ 56563

89 (Italy or Italian Republic).ti,ab. 33860

90 Latvia/ 1451

91 Latvia.ti,ab. 1129

92 Liechtenstein/ 55

93 Liechtenstein.ti,ab. 77

94 Lithuania/ 2202

95 Lithuania.ti,ab. 1591

96 Luxembourg/ 506

97 Luxembourg.ti,ab. 499

98 Malta/ 1148

99 Malta.ti,ab. 1132

100 monaco/ 53

101 Monaco.ti,ab. 68

102 Netherlands/ 30183

103 (Netherlands or Holland).ti,ab. 19935

104 exp Norway/ 12603

105 Norway.ti,ab. 10021

106 Poland/ 24279

107 Poland.ti,ab. 15124

108 exp Portugal/ 8436

109 (Portugal or Portuguese Republic).ti,ab. 5794

110 San Marino/ 39

111 San Marino.ti,ab. 45

112 Scandinavia/ 54823

113 (((Scandinavian or Nordic) adj1 (countr* or state? or region)) or Scandinavia).ti,ab. 1997

114 Slovakia/ 3630

115 (Slovakia or Slovak Republic).ti,ab. 3109

116 Slovenia/ 3642

117 Slovenia.ti,ab. 2437

118 exp Spain/ 43992

119 (Spain or Balearic Islands or Canary Islands).ti,ab. 27820

120 Sweden/ 26774

121 Sweden.ti,ab. 16508

122 Switzerland/ 15106

123 (Switzerland or Swiss Confederation).ti,ab. 9142

124 exp United Kingdom/ or channel islands/ or "isle of man"/ 102705

125 (United Kingdom or UK or Great Britain or GBR or Northern Ireland or Scotland or Channel Islands or Isle of Man or (Wales not New South Wales) or (England not New England)).ti,ab. 74029

126 Moldova/ 739

127 Moldova.ti,ab. 429

128 exp Ukraine/ 3803

129 Ukraine.ti,ab. 2548

130 Albania/ 1192

131 Albania.ti,ab. 1016

132 Armenia/ 839

133 Armenia.ti,ab. 708

134 exp Azerbaijan/ 1323

135 Azerbaijan.ti,ab. 1146

136 Belarus/ 1122

137 (Belarus or Byelarus or Byelorussia or Belorussia).ti,ab. 998

138 exp "Bosnia and Herzegovina"/ 1790

139 (Bosnia or Herzegovina).ti,ab. 1284

140 Bulgaria/ 5409

141 Bulgaria.ti,ab. 3581

142 Georgia.ti,ab. not "georgia (u.s.)"/ 5293

143 Kosovo/ 415

144 Kosovo.ti,ab. 535

145 "Montenegro (republic)"/ 0

146 Montenegro.ti,ab. 786

147 "republic of north macedonia"/ 88

148 North Macedonia.ti,ab. 34

149 Romania/ 9685

150 Romania.ti,ab. 4682

151 exp Russian Federation/ 19042

152 ussr/ 21060

153 (Russia or Russian Federation or USSR or Union of Soviet Socialist Republics or Soviet Union).ti,ab. 18491

154 exp Serbia/ 4078

155 Serbia.ti,ab. 2987

156 "Turkey (republic)"/ 0

157 (Turkey.ti,ab. not "Turkey (bird)"/) or (Anatolia or Asia Minor).ti,ab. 24186

158 45 or 46 or 47 or 48 or 49 or 50 or 51 or 52 or 53 or 54 or 55 or 56 or 57 or 58 or 59 or 60 or 61 or 62 or 63 or 64 or 65 or 66 or 67 or 68 or 69 or 70 or 71 or 72 or 73 or 74 or 75 or 76 or 77 or 78 or 79 or 80 or 81 or 82 or 83 or 84 or 85 or 86 or 87 or 88 or 89 or 90 or 91 or 92 or 93 or 94 or 95 or 96 or 97 or 98 or 99 or 100 or 101 or 102 or 103 or 104 or 105 or 106 or 107 or 108 or 109 or 110 or 111 or 112 or 113 or 114 or 115 or 116 or 117 or 118 or 119 or 120 or 121 or 122 or 123 or 124 or 125 or 126 or 127 or 128 or 129 or 130 or 131 or 132 or 133 or 134 or 135 or 136 or 137 or 138 or 139 or 140 or 141 or 142 or 143 or 144 or 145 or 146 or 147 or 148 or 149 or 150 or 151 or 152 or 153 or 154 or 155 or 156 or 157 622176

159 24 or 43 1281902

160 28 or 44 26703

161 158 and 159 and 160 1542

162 limit 161 to yr="2000 -Current" 1288

**APA PsycInfo** was searched using the OvidSP interface <1806 to July 1 2021>

1 exp *marginalized groups/ or *minority stress/ or *social disadvantage/ 579

2 exp *social exclusion/ 995

3 exp *disadvantaged/ or exp *cultural deprivation/ or exp *economic disadvantage/ or exp *homeless/ or exp *poverty/ or exp *social deprivation/ or exp *socioeconomic status/ 62173

4 *social class/ or *lower class/ 6371

5 *unemployment/ or *employment status/ or *social issues/ 27629

6 exp *poverty/ 7491

7 exp *homeless/ 7166

8 *rural health/ or *communities/ or *health care access/ or *health care delivery/ or *health care services/ or *health disparities/ 77115

9 exp *educational background/ 7445

10 exp *Multiple Disabilities/ or exp *Learning Disabilities/ or exp *Disabilities/ 47035

11 exp *lgbtq/ 26766

12 exp *sexual minority groups/ or *bisexuality/ or *gender nonconforming/ or *homosexuality/ or *intersex conditions/ or *minority stress/ or *transgender/ or *"transgender (attitudes toward)"/ 15264

13 exp *sex work/ 3557

14 *prisoners/ or exp *female criminal offenders/ 9618

15 exp *hiv/ 38744

16 exp *hepatitis/ 2558

17 exp *mental disorders/ 803520

18 exp *Drug Abuse/ or exp *Alcoholism/ or exp *"Substance Use Disorder"/ 115416

19 exp *sexual abuse/ or *child abuse/ or *intimate partner violence/ or *pedophilia/ 52519

20 exp *"Racial and Ethnic Groups"/ or exp *Minority Groups/ 99369

21 exp *Immigration/ 19307

22 exp *refugees/ or exp *asylum seeking/ 5947

23 exp *human trafficking/ 1021

24 1 or 2 or 3 or 4 or 5 or 6 or 7 or 8 or 9 or 10 or 11 or 12 or 13 or 14 or 15 or 16 or 17 or 18 or 19 or 20 or 21 or 22 or 23 1167215

25 (exp *cancer screening/ or *neoplasms/) and *human papillomavirus/ 290

26 (Vulnerab* or stigma* or Underprivilege* or Marginali#e* or Exclu* or "Hard-to-reach" or "Difficult to reach" or Disadvantage* or Underserved or Discriminat* or impoverished or Depriv* or Unemploy* or Poverty or Poor or Homeless* or "low income" or Uneducated or rural* or ((Soci* or economic*) adj3 (status or class or position or depriv*)) or ((Low or poor* or limited) adj3 educat*)).mp. [mp=title, abstract, heading word, table of contents, key concepts, original title, tests & measures, mesh] 663257

27 (Disabilit* or disabled).mp. [mp=title, abstract, heading word, table of contents, key concepts, original title, tests & measures, mesh] 164346

28 (LGBT* or lesbian* or bisexual* or gay or homosexual*).mp. [mp=title, abstract, heading word, table of contents, key concepts, original title, tests & measures, mesh] 47700

29 "sexual minorit*".mp. [mp=title, abstract, heading word, table of contents, key concepts, original title, tests & measures, mesh] 5350

30 ("non-binary" or "gender non-conforming" or "gender fluid" or "gender queer" or genderqueer or transm#n or "trans m#n" or "trans male" or transmasculine or "trans masculine" or transmasc* or "cross dress*" or transgender or transsexual or transvestite* or FTM or "female to male").mp. [mp=title, abstract, heading word, table of contents, key concepts, original title, tests & measures, mesh] 20746

31 ((Sex* adj3 (work* or trade or industry or service*)) or Prostitut* or escort*).mp. [mp=title, abstract, heading word, table of contents, key concepts, original title, tests & measures, mesh] 13869

32 (Detained or Detention or Incarcerat* or Imprison* or prison* or jail* or inmate).mp. [mp=title, abstract, heading word, table of contents, key concepts, original title, tests & measures, mesh] 45359

33 (("human immunodeficiency virus" or HIV) adj3 (positive or living or infection*)).mp. [mp=title, abstract, heading word, table of contents, key concepts, original title, tests & measures, mesh] 25407

34 ((Hepatitis adj3 (B or C)) or Blood-borne virus*).mp. [mp=title, abstract, heading word, table of contents, key concepts, original title, tests & measures, mesh] 4073

35 ((Mental* or Psych*) adj3 (condition* or ill* or disorder* or issue* or diagnos* or problem*)).mp. [mp=title, abstract, heading word, table of contents, key concepts, original title, tests & measures, mesh] 345753

36 (((Substance or alcohol* or drug) adj3 (disorder* or use* or abuse* or misuse or addict* or depend* or problem* or issue*)) or Alcoholi*).mp. [mp=title, abstract, heading word, table of contents, key concepts, original title, tests & measures, mesh] 235109

37 (Sexual* adj3 (abuse* or assault* or trauma*)).mp. [mp=title, abstract, heading word, table of contents, key concepts, original title, tests & measures, mesh] 38961

38 (ethnic* or minorit* or BME or BAME or race or racial or caste or indian* or pakistani* or chinese or arab* or bangladeshi* or gypsy or gypsies or traveller* or asian* or black or african* or caribbean or "central and eastern european*" or "central european*" or "eastern european*" or iraqi* or somali* or bosnian* or serbian* or brazilian* or surinamese or "north african*" or "south american*" or romanian* or roma* or ex-yugoslavia or turkish or albanian* or russian*).mp. [mp=title, abstract, heading word, table of contents, key concepts, original title, tests & measures, mesh] 450436

39 (traffick* or refugee* or immigra* or migra* or foreigner* or "asylum-seek*").mp. [mp=title, abstract, heading word, table of contents, key concepts, original title, tests & measures, mesh] 87688

40 26 or 27 or 28 or 29 or 30 or 31 or 32 or 33 or 34 or 35 or 36 or 37 or 38 or 39 1642877

41 ((Cervical or Pap or Papanicolaou or HPV or "high-risk HPV" or hrHPV or "Human Papillomavirus" or smear) adj5 (screen* or test* or smear)).mp. [mp=title, abstract, heading word, table of contents, key concepts, original title, tests & measures, mesh] 2634

42 europe/ or eastern europe/ or southern europe/ or western europe/ 0

43 balkan peninsula/ or baltic states/ or vatican city state/ or Gibraltar/ 0

44 (europe or balkan*or mediterranean or vatican or gibraltar).ti,ab. 21988

45 Andorra/ 0

46 Andorra.ti,ab. 13

47 Austria/ 0

48 Austria.ti,ab. 2544

49 Baltic States/ 0

50 (Baltic adj1 (state? or countr* or region)).ti,ab. 104

51 exp Belgium/ 0

52 Belgium.ti,ab. 3955

53 benelux/ 0

54 Benelux.ti,ab. 11

55 Croatia/ 0

56 Croatia.ti,ab. 1459

57 Czech Republic/ 0

58 Czechoslovakia/ 0

59 (Czech Republic or Czechia or Czechoslovakia).ti,ab. 1891

60 Cyprus/ 0

61 Cyprus.ti,ab. 1063

62 Denmark/ 0

63 Denmark.ti,ab. 5488

64 Estonia/ 0

65 Estonia.ti,ab. 879

66 faroe islands/ 0

67 Faroe Islands.ti,ab. 68

68 exp Finland/ 0

69 Finland.ti,ab. 7045

70 exp France/ 0

71 (France or French Republic).ti,ab. 12314

72 exp Germany/ 0

73 Germany.ti,ab. 20925

74 Gibraltar/ 0

75 Gibraltar.ti,ab. 48

76 Greece/ 0

77 (Greece or Hellenic Republic).ti,ab. 4462

78 Greenland/ 0

79 Greenland.ti,ab. 201

80 Hungary/ 0

81 Hungary.ti,ab. 2130

82 Iceland/ 0

83 Iceland.ti,ab. 1089

84 Ireland/ 0

85 ((Ireland or Eire) not Northern Ireland).ti,ab. 5336

86 exp Italy/ 0

87 (Italy or Italian Republic).ti,ab. 11369

88 Latvia/ 0

89 Latvia.ti,ab. 297

90 Liechtenstein/ 0

91 Liechtenstein.ti,ab. 19

92 Lithuania/ 0

93 Lithuania.ti,ab. 693

94 Luxembourg/ 0

95 Luxembourg.ti,ab. 292

96 Malta/ 0

97 Malta.ti,ab. 297

98 monaco/ 0

99 Monaco.ti,ab. 32

100 Netherlands/ 0

101 (Netherlands or Holland).ti,ab. 15665

102 exp Norway/ 235

103 Norway.ti,ab. 7955

104 Poland/ 0

105 Poland.ti,ab. 3880

106 exp Portugal/ 0

107 (Portugal or Portuguese Republic).ti,ab. 3221

108 San Marino/ 0

109 San Marino.ti,ab. 17

110 Scandinavia/ 0

111 (((Scandinavian or Nordic) adj1 (countr* or state? or region)) or Scandinavia).ti,ab. 1777

112 Slovakia/ 0

113 (Slovakia or Slovak Republic).ti,ab. 709

114 Slovenia/ 0

115 Slovenia.ti,ab. 908

116 exp Spain/ 0

117 (Spain or Balearic Islands or Canary Islands).ti,ab. 13134

118 Sweden/ 0

119 Sweden.ti,ab. 12338

120 Switzerland/ 0

121 (Switzerland or Swiss Confederation).ti,ab. 4594

122 exp United Kingdom/ or channel islands/ or "isle of man"/ 0

123 (United Kingdom or UK or Great Britain or GBR or Northern Ireland or Scotland or Channel Islands or Isle of Man or (Wales not New South Wales) or (England not New England)).ti,ab. 71480

124 Moldova/ 0

125 Moldova.ti,ab. 150

126 exp Ukraine/ 0

127 Ukraine.ti,ab. 870

128 Albania/ 0

129 Albania.ti,ab. 256

130 Armenia/ 0

131 Armenia.ti,ab. 259

132 exp Azerbaijan/ 0

133 Azerbaijan.ti,ab. 171

134 Belarus/ 0

135 (Belarus or Byelarus or Byelorussia or Belorussia).ti,ab. 238

136 exp "Bosnia and Herzegovina"/ 0

137 (Bosnia or Herzegovina).ti,ab. 834

138 Bulgaria/ 0

139 Bulgaria.ti,ab. 711

140 exp "Georgia (republic)"/ 0

141 Georgia.ti,ab. not "georgia (u.s.)"/ 4100

142 Kosovo/ 0

143 Kosovo.ti,ab. 374

144 "Montenegro (republic)"/ 0

145 Montenegro.ti,ab. 89

146 "republic of north macedonia"/ 0

147 North Macedonia.ti,ab. 9

148 Romania/ 0

149 Romania.ti,ab. 1418

150 exp Russian Federation/ 0

151 ussr/ 0

152 (Russia or Russian Federation or USSR or Union of Soviet Socialist Republics or Soviet Union).ti,ab. 7857

153 exp Serbia/ 0

154 Serbia.ti,ab. 823

155 "Turkey (republic)"/ 0

156 (Turkey.ti,ab. not "Turkey (bird)"/) or (Anatolia or Asia Minor).ti,ab. 7935

157 or/42-156 [ALL COUNTRIES IN EUROPE] 222332

158 24 or 40 2104939

159 25 or 41 2774

160 157 and 158 and 159 133

161 limit 160 to (human and yr="2000 -Current")
